# Supplementary material for: Peroxisomal very long-chain fatty acid transport is targeted by herpesviruses and the antiviral host response
Source: Commun Biol. 2022 Sep 9;5:944. doi: 10.1038/s42003-022-03867-y (PMC9462615; doi:10.1038/s42003-022-03867-y)
Supplement: Supplementary file 2 — Supplementary Information [file 42003_2022_3867_MOESM2_ESM.pdf]

SUPPLEMENTARY INFORMATION

Weinhofer et al. “Peroxisomal very long-chain fatty acid transport is targeted by herpesviruses and the antiviral host response”

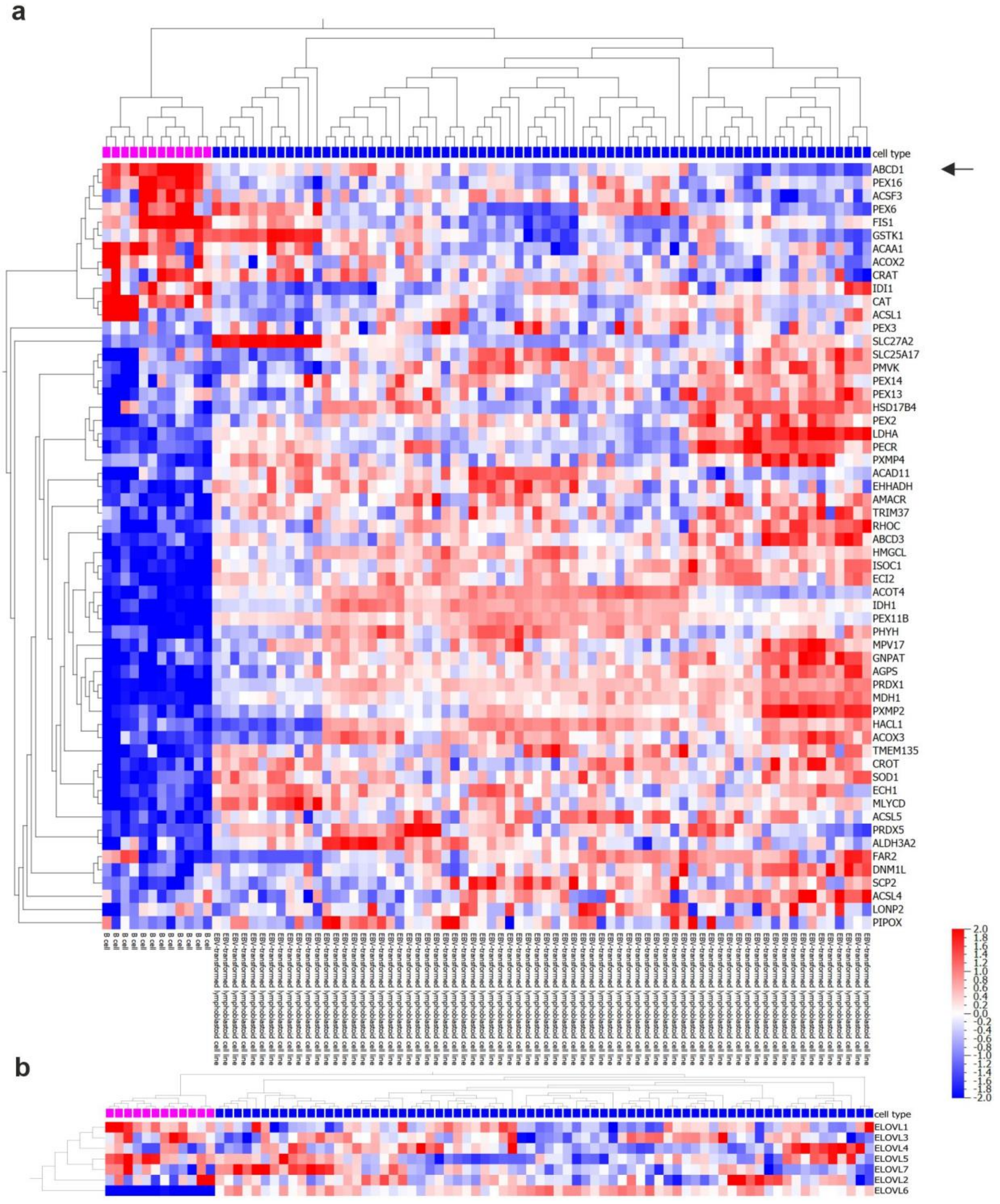

c

Peroxisomal genes:

**Gene symbol**	***P*-value**	**Log2 Fold Change**
ABCD1	5.80E-16	-0.91
ABCD3	8.81E-12	0.43
ACAA1	1.14E-04	-0.27
ACAD11	9.08E-10	0.69
ACOT4	2.25E-18	1.79
ACOX2	2.54E-06	-0.26
ACOX3	3.49E-11	0.65
ACSF3	6.80E-03	-0.16
ACSL1	1.07E-02	-0.49
ACSL4	3.11E-02	0.10
ACSL5	1.32E-12	0.41
AGPS	1.57E-20	0.87
ALDH3A2	5.18E-07	0.45
AMACR	3.14E-13	0.39
CAT	4.85E-17	-0.91
CRAT	3.66E-02	-0.14
CROT	1.01E-13	0.53
DNM1L	2.68E-07	0.49
ECH1	7.11E-17	0.69
ECI2	4.25E-23	1.30
EHHADH	1.41E-13	0.43
FAR2	3.10E-03	0.40
FIS1	1.37E-11	-0.72
GNPAT	3.44E-13	0.52
GSTK1	8.67E-03	-0.35
HACL1	3.09E-10	0.80
HMGCL	1.38E-27	1.06
HSD17B4	1.31E-04	0.32
IDH1	1.59E-28	2.33
IDI1	1.15E-04	-0.69
ISOC1	1.51E-22	1.05
LDHA	1.05E-07	0.83
LONP2	2.32E-02	0.11
MDH1	1.39E-30	1.52
MLYCD	3.66E-17	0.56
MPV17	1.68E-12	0.53
PECR	6.72E-09	0.78
PEX11B	1.90E-42	1.92
PEX13	1.97E-04	0.15
PEX14	1.61E-10	0.48
PEX16	2.15E-15	-0.52
PEX2	1.79E-11	0.38
PEX3	2.82E-02	0.09
PEX6	4.54E-03	-0.36
PHYH	2.05E-15	0.81
PIPOX	1.86E-03	0.14
PMVK	6.09E-06	0.44
PRDX1	5.17E-36	2.06

PRDX5	1.80E-10	0.55
PXMP2	3.84E-12	1.08
PXMP4	3.47E-07	0.23
RHOC	6.24E-13	0.69
SCP2	8.34E-07	0.21
SLC25A17	6.96E-05	0.31
SLC27A2	1.44E-04	0.74
SOD1	1.68E-18	0.61
TMEM135	1.02E-14	0.72
TRIM37	4.29E-10	0.42

Genes encoding enzymes involved in LCFA and VLCFA synthesis:

**Gene symbol**	***P*-value**	**Log2 Fold Change**
ELOVL1	1.76E-04	-0.25
ELOVL2	5.29E-01	-0.03
ELOVL3	3.78E-02	-0.08
ELOVL4	1.83E-01	0.10
ELOVL5	5.83E-05	-0.58
ELOVL6	4.40E-35	1.68
ELOVL7	7.91E-01	0.01

Supplementary Figure 1. Effect of EBV transformation on peroxisome related genes and expression of enzymes involved in LCFA and VLCFA synthesis. (a-b) mRNA levels from primary B cells derived from six healthy donors and their corresponding EBV-immortalized B lymphocytes were retrieved from the Gene Expression Omniubs database file GSE26212 and analyzed using the Qlucore Omics Explorer Software (Qlucore AB, Lund, Sweden). The heatmaps showing (a) significantly dysregulated peroxisomal genes (Two-Group comparison test, $P \leq 0.05$) and (b) genes encoding enzymes involved in LCFA and VLCFA synthesis were generated using the median value of all probes for the specific genes and scaled with Z-score. The transcriptomics data were generated by Caliskan *et al.* [1] by hybridization of the cDNAs to HumanHT-13 v3 Expression BeadChip arrays (Illumina Inc.). B cells are shown in two technical replicates; for the corresponding EBV-immortalized B lymphocytes, six independent cell lines were generated from each individual and analyzed in two technical replicates. The intensity estimates of the transcriptomics data were log-transformed and the quantile normalized using the “lumi” package in R v2.10.1 by Caliskan et al. *ABCD1* is indicated by an arrow. *P*-values and log2 fold changes of the transcriptomics data are shown in **(c)**.

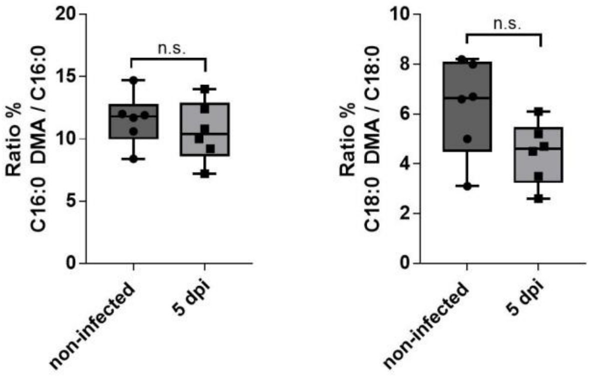

Supplementary Figure 2. Analysis of the plasmalogen content in primary B cells at day 5 post EBV infection. Plasmalogen levels were measured as their dimethylacetal (DMA) derivatives by GC-MS in lipid extracts of primary B cells isolated from six healthy donors. Part of the cells were *in vitro* infected with EBV and harvested 5 days post infection (dpi). The relative amount of plasmalogens was estimated as the ratio of C16:0 DMA to C16:0 and of C18:0 DMA to C18:0. The data are depicted as boxplots (median ± interquartile range). *n.s.* = not significant (two-tailed paired Student's *t*-test).

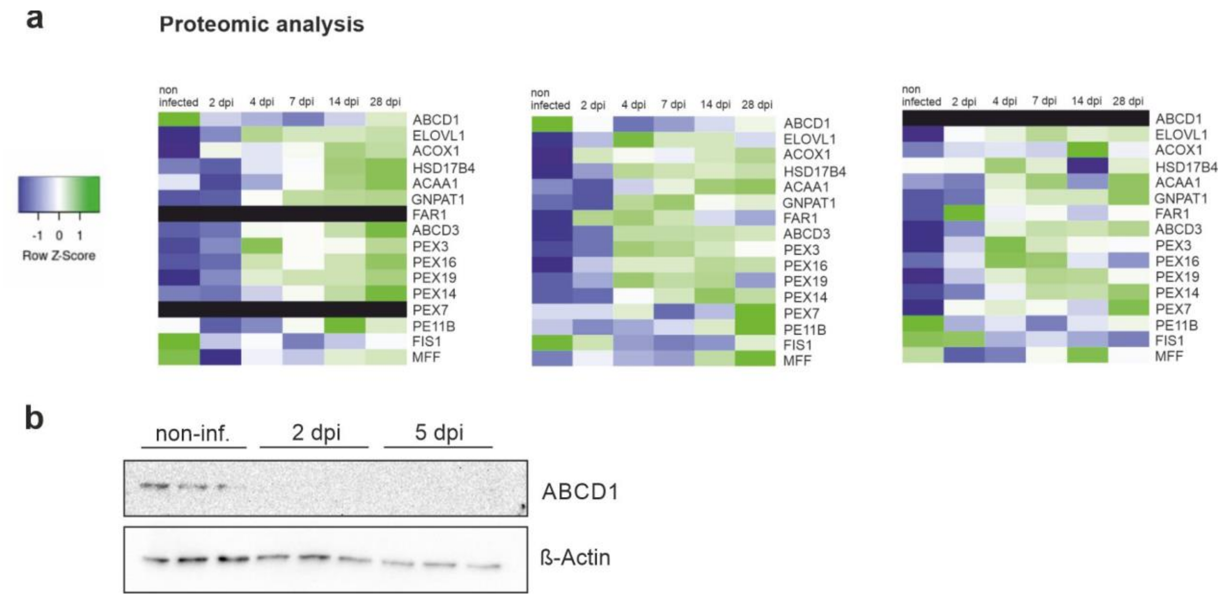

Supplementary Figure 3. Temporal analysis of peroxisomal proteins and enzymes involved in VLCFA synthesis in primary human B cells upon EBV infection. (a) Time-resolved multiplexed tandem mass tag-based proteomics data was retrieved from Wang *et al* [2] (http://www.proteomexchange.org/; PXD013034). For each of the three replicates (whole cell lysates WCL1-3), B cells were isolated from four human donors. Black bars, data not available. **(b)** Western Blot analysis (own data) to confirm the downregulation of ABCD1 protein levels upon EBV infection. Protein extracts were prepared from primary human B cells derived from a healthy donor before *in vitro* infection with EBV and at 2 and 5 days post infection (dpi), (n=1, 3 technical replicates per condition). Uncropped western blot images are shown in Supplementary Figure 8.

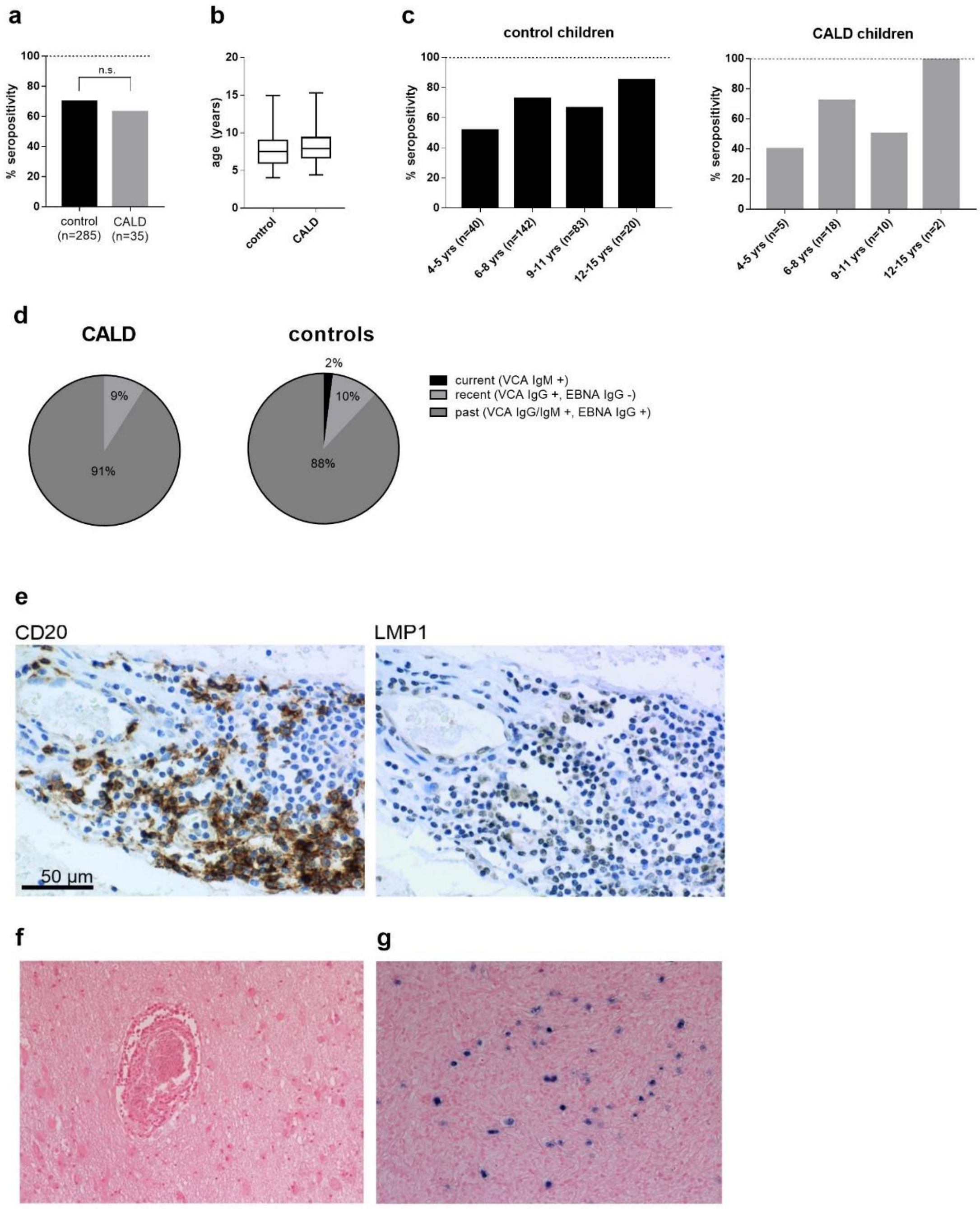

Supplementary Figure 4. EBV positivity is not elevated in serum and post-mortem brain tissue of children with cerebral ALD. (a-d) Sera from children with proven CALD before allogeneic stem cell transplantation (*n*=35, age range= 4-15 years, median age=8 years) and from controls with conditions unrelated to X-ALD (*n*=285, age range= 4-15 years, median age=8 years) were tested for EBV seropositivity using antibodies directed against the viral

capsid antigen (VCA) IgG and IgM; n.s.= non significant (one-sided Fisher´s exact test) (a). The distribution of age within the group of controls and CALD patients is shown in (b). The data are depicted as boxplots (median ± interquartile range). In (c), both control and CALD groups are splitted up according to age and the EBV positivity is shown for each of these age subgroups. (d) The positive samples were assessed for past, acute or recent EBV infections using antibodies directed against VCA IgG, VCA IgM and EBV nuclear antigen (EBNA)-1 IgG (B). **(e)** Immunohistochemistry for the B cell marker CD20 and the EBV protein LMP1 (latent membrane protein 1) and **(f, g)** in situ hybridization for the EBV-associated small RNA EBER was performed on post-mortem brain tissue of 4 CALD cases. One representative case with B cell infiltration of perivascular cuffs is shown (e, f). No positive staining was detected for either LMP1 or EBER. For the EBER in situ hybridization, post-mortem brain tissue of a case with lymphomatoid granulomatosis served as positive control (g).

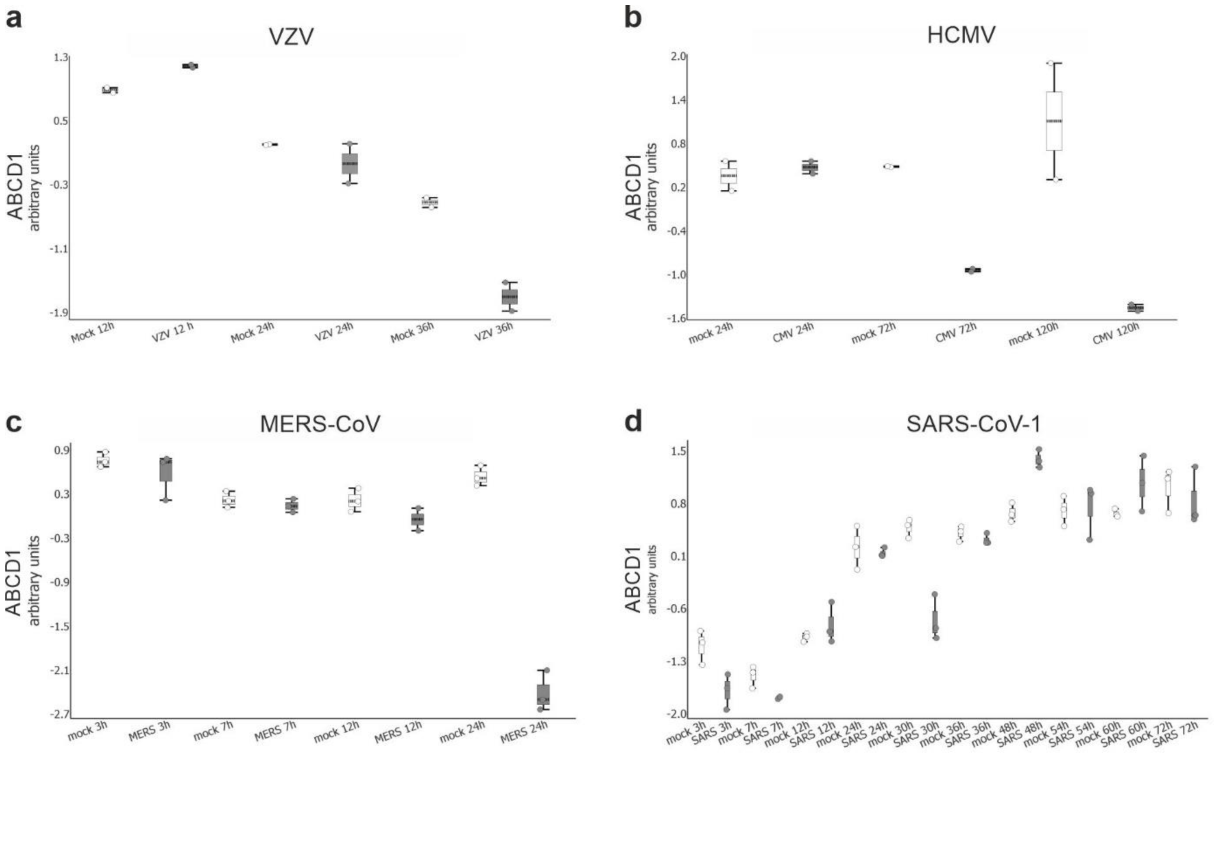

Supplementary Figure 5. Targeting of *ABCD1* expression by herpes- and coronaviruses at different time points. Time resolved transcriptomics datasets from human cells infected *in vitro* with herpes- and coronaviruses were retrieved from Gene Expression Omnibus database files and analyzed for *ABCD1* expression before and at different time points after infection. **(a)**

VZV-infected melanoma cells (two biological replicates, GSE85493); **(b)** HCMV-infected lung fibroblasts (two biological replicates, GSE99454); **(c)** MERS-CoV-infected bronchial epithelial cells (three replicates, GSE45042); **(d)** SARS-CoV-1-infected bronchial epithelial cells (three replicates, GSE33267). The data are depicted as boxplots (median ± interquartile range). Arbitrary units on the y-axis represent normalized variable values that were obtained using the defaults to Z-score normalization in the Qlucore Software 3.5 (mean zero and standard deviation 1).

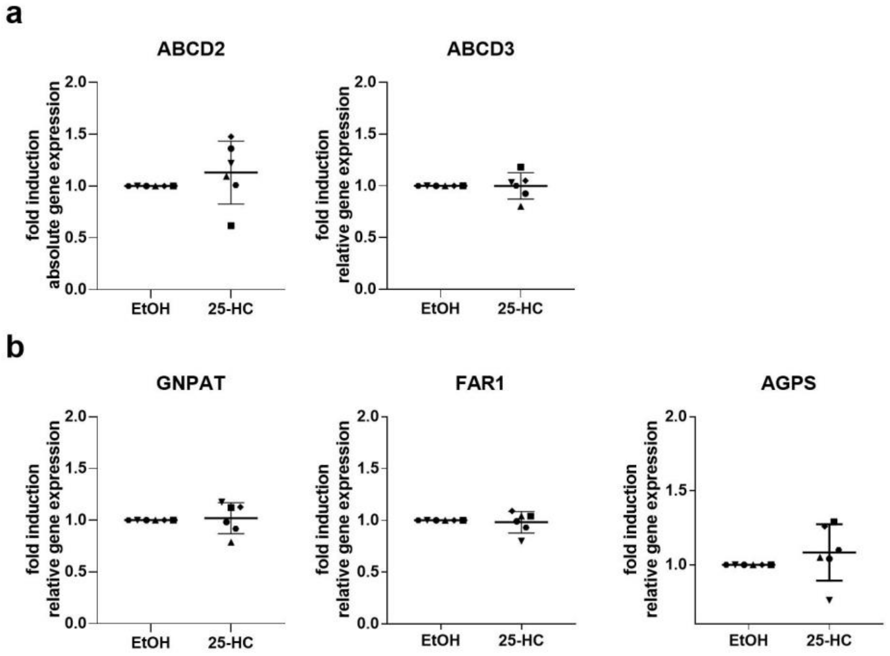

Supplementary Figure 6. Expression of the peroxisomal fatty acid transporters *ABCD2* and *ABCD3* and enzymes for plasmalogen synthesis is not affected by 25-hydroxycholesterol. EBV-immortalized B lymphocytes derived from healthy donors (*n* = 6) were treated with 2 µM 25-hydroxycholesterol (25-HC) or the solvent (ethanol, EtOH) for 24 h before RNA was isolated and RT-qPCR carried out for **(a)** the peroxisomal ABC transporter genes *ABCD2* and *ABCD3* and **(b)** genes (*GNPAT*, *FAR1* and *AGPS*) for the peroxisomal enzymes of plasmalogen synthesis. The mean is indicated by a horizontal line ± S.D. Data were normalized to *HPRT* mRNA levels and are displayed as fold induction over EtOH-treated cells.

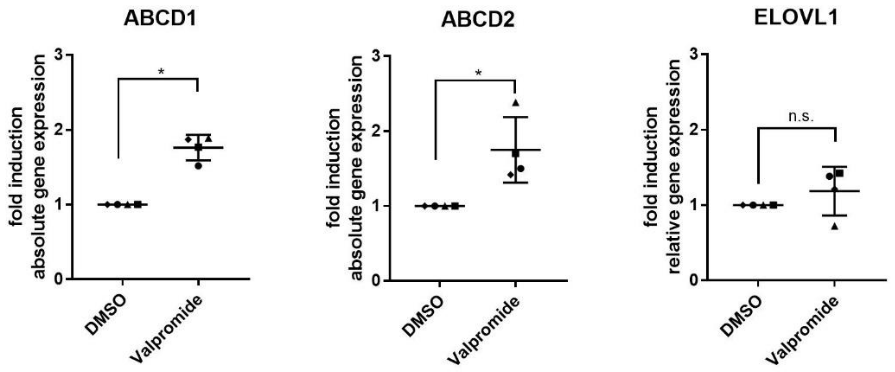

Supplementary Figure 7. The EBV lytic cycle inhibitor valpromide stimulates *ABCD1* and *ABCD2* expression. EBV-immortalized B lymphocytes derived from healthy donors (*n*=4) were treated with 10 mM valpromide or the solvent DMSO for 24 h before RNA was isolated and RT-qPCR carried out for *ABCD1*, *ABCD2* and *ELOVL1* expression. Data were normalized to *HPRT* mRNA levels and are displayed as fold induction over DMSO-treated cells. The mean is indicated by a horizontal line ± S.D. For statistical analysis, the raw values used to generate the fold-change display were used (two-tailed paired Student's *t*-test, *$P \leq 0.05$).

a

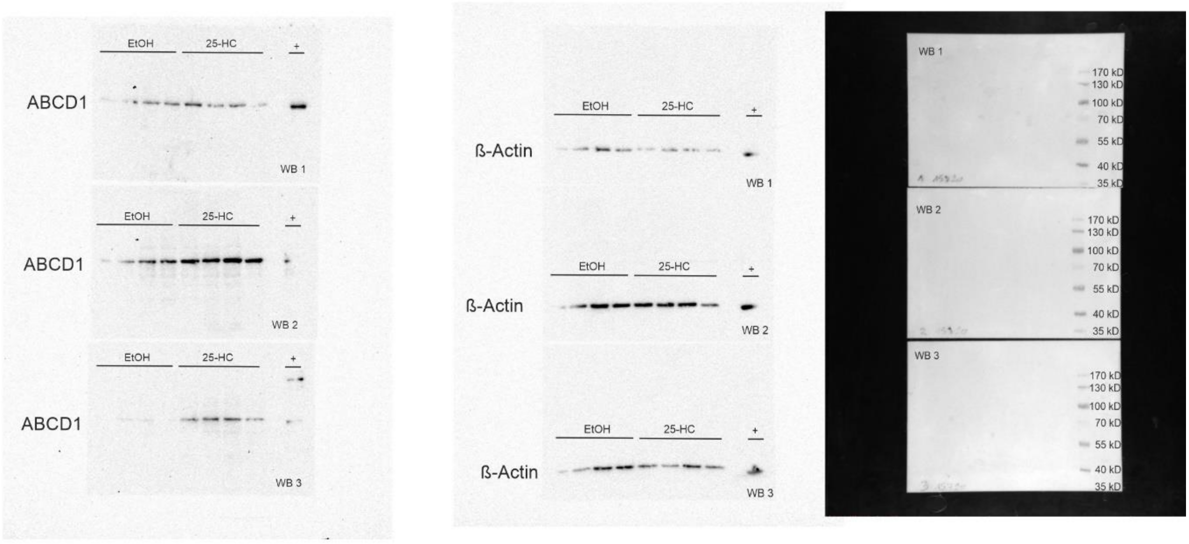

b

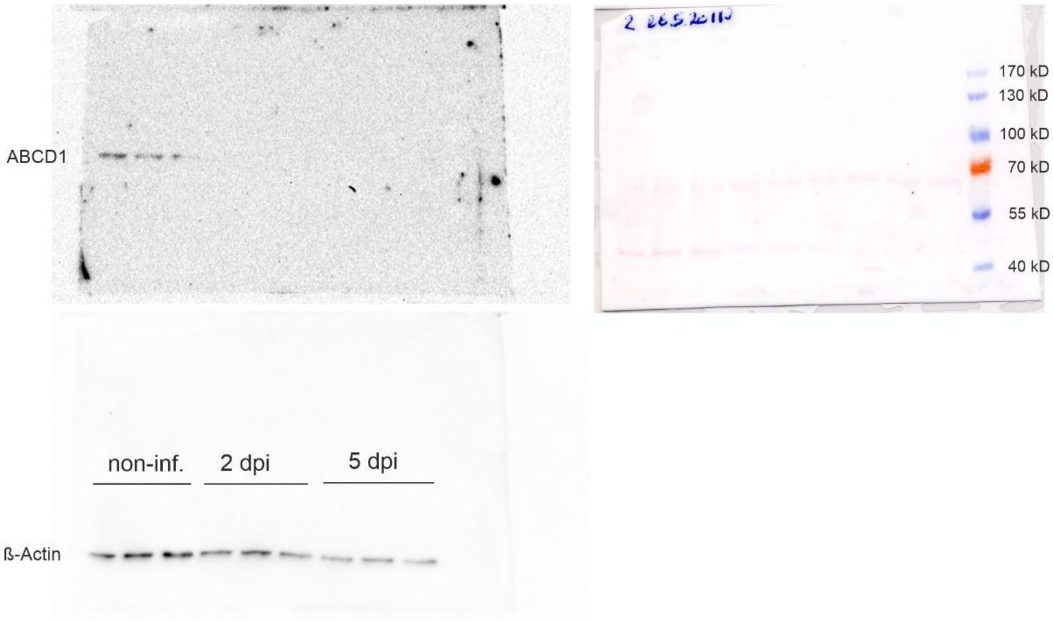

Supplementary Figure 8. Unedited/Uncropped western blot images of primary B cells and EBV-immortalized B lymphocytes derived from healthy controls. (a) Unedited/uncropped western blot images to Fig. 5d. **(b)** Unedited/uncropped western blot images to Supplementary Figure 3b. (+ human skin fibroblast cell pellet).

Supplementary Table 1. Prediction analysis for miR-9-5p targeting *ABCD1*

Database	Number of predicted miR-9-5p target sites	Nt-Position after the stop codon	Score
TargetScan v7.0<br>https://www.targetscan.org/vert_70/	2	533-539;<br>862-868	Context++ score: -0.03<br>Context++ score: -0.12
PicTar<br>https://pictar.mdc-berlin.de/	2	533, 862	PicTar score: 4.43
RNA22 v2<br>https://cm.jefferson.edu/rna22/Interactive/	2	421-439;<br>847-868	*p*-value: 0.043<br>*p*-value: 0.026
DIANA-microT-CDS v5.0<br>https://dianalab.e-ce.uth.gr/	3	388-405<br>523-538<br>843-867	Score: 0.0025<br>Score: 0.0052<br>Score: 0.0330
mirCode11<br>http://www.mircode.org/	2	533-538; 862-867	-
miRDB<br>http://mirdb.org/	2	533, 862	Target score: 69

Five bioinformatic miRNA target prediction algorithms (TargetScan v7.0 [3-6], PicTar [7], DIANA-microT-CDS v5.0 [8,9], mirCode 11 [10] and miRDB [11,12]) predicted the same two miR-9-5p target sites located at nucleotide positions 533-538 and 862-868 after the stop codon within the 3'-UTR of ABCD1. The second site, at position 862-868, was also predicted by the RNA22 v2 [13] algorithm, which also identifyed an additional putative site at position 421-439. Next to these two sites at nucleotide positions 533-538 and 862-868, the DIANA-microT-CDS vs5.0 algorithm predicted an additional miR-9-5p target site at position 388-405.

Supplementary Table 2. Prediction analysis for miR-155 targeting *ABCD2*

Database	Number of predicted miR-155 target sites	Nt-Position after the stop codon	Score
TargetScan v7.0<br>https://www.targetscan.org/vert_70/	1	128-134	Context++score: -0.02
microCosm targets v5<br>https://www.ebi.ac.uk	1	128-134	Rank metric score: 4,86
miRCode 11<br>http://www.mircode.org/	1	128-134	-
miRanda-mirSVR	3	128-134;<br>177-182;<br>195-199	mirSVR score: -1.126<br>mirSVR score: -0.039<br>mirSVR score: -0.456
miRDB<br>http://mirdb.org/	1	128	Target score: 75
RNA22 v2<br>https://cm.jefferson.edu/rna22/Interactive/	-		
PicTar<br>https://pictar.mdc-berlin.de/	-		

Five bioinformatic miRNA target prediction algorithms (TargetScan v7.0 [3-6], microCosm targets v5 [14], miRCode 11 [10], miRanda-mirSVR [13] and miRDB [11,12] predicted the same miR-155 target site located at nucleotide position 128-135 after the stop codon within the 3'-UTR of *ABCD2*. The miRanda-mirSVR [13] algorithm predicted two additional miR-155 target sites in the human *ABCD2* 3'-UTR. The RNA22 v2 [13] and PicTar [7] algorithms did not identify any

putative miR-155 target site within the 3'-UTR of *ABCD2*.

Supplementary Table 3. *P*-values and Log2 fold changes for *ABCD1* expression from transcriptomic datasets shown in Fig. 4

Data set	virus	*P*-value	Log2 Fold Change	Number of replicates per condition
GSE129582	HSV-1	8,88E-03	-1.80	2
GSE85493	VZV	3,02E-02	-0.37	2
GSE135644	EBV	1,75E-05	-0.48	5
GSE99454	HCMV	3,87E-04	-0.85	2
GSE149808	Roseolovirus	N.A.	N.A.	1
GSE27136	KSHV	3,77E-01	-0.07	3
GSE45042	MERS-CoV	8,13E-05	-2.13	3
GSE33267	SARS-CoV-1	3,55E-02	-0.24	3
GSE150392	SARS-CoV-2	1,03E-01	-0.69	3
CRA002390	SARS-CoV-2	6.96E-06*	1.38*	3

**P*-value and log2 fold change were obtained from Xiong et al. [15], Supplementary File 1 .

Supplementary Table 4. Primers used for RT-qPCR and EBV genome copy number analysis

Gene	Accession number, mRNA	Product length (bp)	Sequence
HPRT	NM_000194	220	F 5´-ccctggcgtcgtgattagt-3´<br>R 5´-caggtcagcaaagaatttatagcc-3`<br>FAM-caggactgaacgtcttgctcgaga-BHQ1
ABCD1	NM_000033	169	F 5´-gagaacatccccatcgtc-3´<br>R 5´-tgtagagcacaccaccgta-3´
ABCD2	NM_005164	79	F 5´-tcctacacaatgtccatctct-3´<br>R 5´-aggacatctttccagtcca-3´<br>Cy5-caaagagaaggaggatgggatgc-BHQ2
ABCD3	M81182	98	F 5´-cggctcatcacaaacagtga-3´<br>R 5´-aggtgttccaccagttttcg-3´
EBNA1	NC_007605	217	F 5´-gccggtgtgttcgtatatgg-3´<br>R 5´-ccttcaaaacctcagcaaatatatga-3´
BHRF1	NC_007605	208	F 5´-ggagatactgttagccctg-3´<br>R 5´-gtgtgttataaatctgttccaag-3´
GNPAT	NM_014236	176	F 5´-tcagaaactacaaagagccatcca-3´<br>R 5´-ttcgtagcagctcaccaacc-3´
FAR1	NM_032228	243	F 5´-gacagacaccacaagagcga-3´<br>R 5´-tcacatttaactgaacagcatctctta-3´

AGPS	NM_003659	131	F 5´-agggaaggaatgtttgagcga-3´<br>R 5´-acacttgttcctccaccaattg-3´
ELOVL1	NM_022821	279	F 5´-attgagctgatggacacagtgat-3´<br>R 5´-gaccaggacaaactggatcagc-3´
ELOVL3	NM_152310	64	F 5´- agcaaggtcatagaactcggagac-3´<br>R 5´- taaagatgagtggccgcttacg-3´
ELOVL7	NM_024930	77	F 5´- ggccagcctaccagaagtatttg-3´<br>R 5´- ggcgacaataacaaactggacaag-3´
BALF5	MK_540419 (EBV genome)	193	F 5´-agcttgatgacgatgccaca-3´<br>R 5´-aggatggaaagggcatgtgg-3´
BZLF1	MK973062 (EBV Genome)	113	F 5´-ctgttgtggtttccgtgtgc-3´<br>R 5´-cagtggtgtttgcttgccc-3´

Supplementary Table 5. Primers used for cloning and *in vitro* mutagenesis of the *ABCD1*- and *ABCD2*-3'-UTR

Gene (mutation)	Accession number	Sequence
ABCD1	NM_000033.3	F 5´-gagctctgaagacacacagcagcatc-3´<br>R 5´-gtcgacgaatagaaagcattcagaaaacatttt-3´
ABCD1_M-TS1	NM_000033.3	F 5´-ctcccccacaagagccctgacaatcccacgggagaggg-3´<br>R 5´-cgtgggattgtcagggctcttgtgggggagaggatgcagg-3´
ABCD1_M-TS2	NM_000033.3	F 5´-ccagctcagtgatagcgaggggtcaccagggggagctgtc-3´<br>R 5´-cctggtgacccctcgctatcactgagctgggaacatggtg-3´
ABCD1_MΔ-TS1	NM_000033.3	F 5´-cctcacctgcatccttcccacgggagaggggagagggacg-3´<br>R 5´-cctctcccgtgggaaggatgcaggtgaggcgctctgtgtg-3´
ABCD1_MΔ-TS2	NM_000033.3	F 5´-gttcccagctcagtgggggtcaccagggggagctgtcctgcg-3´<br>R 5´-gctccccctggtgacccccactgagctgggaacatggtgtcc-3´
ABCD2	AJ000327.1	F 5´-ggtgtcgacggagaagactcagtgctgaa-3´<br>R 5´-ctgcggccgccaatgtggtatcacaggcagc-3´
ABCD2_M-TS1	AJ000327.1	F 5´-gacatgttttataagattgctgatcaaaggaagtatatgat-3´<br>R 5´-cttcctttgatcagcaatcttataaaacatgtcttgctgcc-3´
ABCD2_M-TS2	AJ000327.1	F 5´-cttttcagaagaaaataaacaaatgatcagtgtaaggtcag-3´<br>R 5´-gtcataatgactgaccttacactgatcatttgttttc-3´
ABCD2_M-TS3	AJ000327.1	F 5´-ggtcagctcggctgacttatactaattcctagtgaagg-3´<br>R 5´-ggaattagtataagtcagccgagctgaccttacataatg-3´
ABCD2_M-TS1/TS2/TS3	AJ000327.1	F 5´-actaattcctagtgaaggcctaatgcac-3´<br>R 5´-tctctgtgcttagcttaacatacttcatgc-3´
ABCD2_ MΔTS1/TS2/TS3	AJ000327.1	F 5´-actaattcctagtgaaggcctaatgcac-3´<br>R 5´-tctctgtgcttagcttaacatacttcatgc-3´
Actin	BC014861.1	F 5´-aactcgagcaaatgcttctaggcggact-3´<br>R 5´-aagagctcaaggtgtgcacttttattcaac-3´

SUPPLEMENTAL REFERENCES

1. Caliskan M, Cusanovich DA, Ober C, Gilad Y. The effects of EBV transformation on gene expression levels and methylation profiles. *Hum Mol Genet.* Apr 15 2011;20(8):1643-1652.
2. Wang LW, Shen H, Nobre L, et al. Epstein-Barr-Virus-Induced One-Carbon Metabolism Drives B Cell Transformation. *Cell metabolism.* Sep 3 2019;30(3):539-555 e511.
3. Agarwal V, Bell GW, Nam JW, Bartel DP. Predicting effective microRNA target sites in mammalian mRNAs. *Elife.* Aug 12 2015;4.
4. Lewis BP, Burge CB, Bartel DP. Conserved seed pairing, often flanked by adenosines, indicates that thousands of human genes are microRNA targets. *Cell.* Jan 14 2005;120(1):15-20.

5. Garcia DM, Baek D, Shin C, Bell GW, Grimson A, Bartel DP. Weak seed-pairing stability and high target-site abundance decrease the proficiency of lsy-6 and other microRNAs. *Nat Struct Mol Biol.* Sep 11 2011;18(10):1139-1146.
6. Grimson A, Farh KK, Johnston WK, Garrett-Engele P, Lim LP, Bartel DP. MicroRNA targeting specificity in mammals: determinants beyond seed pairing. *Mol Cell.* Jul 6 2007;27(1):91-105.
7. Krek A, Grun D, Poy MN, et al. Combinatorial microRNA target predictions. *Nat Genet.* May 2005;37(5):495-500.
8. Paraskevopoulou MD, Georgakilas G, Kostoulas N, et al. DIANA-microT web server v5.0: service integration into miRNA functional analysis workflows. *Nucleic Acids Res.* Jul 2013;41(Web Server issue):W169-173.
9. Reczko M, Maragkakis M, Alexiou P, Grosse I, Hatzigeorgiou AG. Functional microRNA targets in protein coding sequences. *Bioinformatics.* Mar 15 2012;28(6):771-776.
10. Jeggari A, Marks DS, Larsson E. miRcode: a map of putative microRNA target sites in the long non-coding transcriptome. *Bioinformatics.* Aug 1 2012;28(15):2062-2063.
11. Chen Y, Wang X. miRDB: an online database for prediction of functional microRNA targets. *Nucleic Acids Res.* Jan 8 2020;48(D1):D127-D131.
12. Liu W, Wang X. Prediction of functional microRNA targets by integrative modeling of microRNA binding and target expression data. *Genome biology.* Jan 22 2019;20(1):18.
13. Miranda KC, Huynh T, Tay Y, et al. A pattern-based method for the identification of MicroRNA binding sites and their corresponding heteroduplexes. *Cell.* Sep 22 2006;126(6):1203-1217.
14. Griffiths-Jones S. miRBase: the microRNA sequence database. *Methods Mol Biol.* 2006;342:129-138.
15. Xiong Y, Liu Y, Cao L, et al. Transcriptomic characteristics of bronchoalveolar lavage fluid and peripheral blood mononuclear cells in COVID-19 patients. *Emerg Microbes Infect.* Dec 2020;9(1):761-770.
