# Supplementary material for: Peroxisomal very long-chain fatty acid transport is targeted by herpesviruses and the antiviral host response
Source: Commun Biol. 2022 Sep 9;5:944. doi: 10.1038/s42003-022-03867-y (PMC9462615; doi:10.1038/s42003-022-03867-y)
Supplement: Supplementary file 3 — Description of Additional Supplementary Files [file 42003_2022_3867_MOESM3_ESM.pdf]

Description of Additional Supplementary Files

File name: Supplementary Data

Description: The source data behind the graphs in the paper
