# Supplementary material for: Peroxisomal very long-chain fatty acid transport is targeted by herpesviruses and the antiviral host response
Source: Commun Biol. 2022 Sep 9;5:944. doi: 10.1038/s42003-022-03867-y (PMC9462615; doi:10.1038/s42003-022-03867-y)
Supplement: Supplementary file 5 — Reporting Summary [file 42003_2022_3867_MOESM5_ESM.pdf]

nature portfolio

Corresponding author(s): Isabelle Weinhofer, Johannes Berger

Last updated by author(s): Aug 16, 2022

# Reporting Summary

Nature Portfolio wishes to improve the reproducibility of the work that we publish. This form provides structure for consistency and transparency in reporting. For further information on Nature Portfolio policies, see our Editorial Policies and the Editorial Policy Checklist.

## Statistics

For all statistical analyses, confirm that the following items are present in the figure legend, table legend, main text, or Methods section.

| n/a | Confirmed | |
|---|---|---|
| ☐ | ☒ | The exact sample size ($n$) for each experimental group/condition, given as a discrete number and unit of measurement |
| ☐ | ☒ | A statement on whether measurements were taken from distinct samples or whether the same sample was measured repeatedly |
| ☐ | ☒ | The statistical test(s) used AND whether they are one- or two-sided<br>*Only common tests should be described solely by name; describe more complex techniques in the Methods section.* |
| ☒ | ☐ | A description of all covariates tested |
| ☐ | ☒ | A description of any assumptions or corrections, such as tests of normality and adjustment for multiple comparisons |
| ☐ | ☒ | A full description of the statistical parameters including central tendency (e.g. means) or other basic estimates (e.g. regression coefficient) AND variation (e.g. standard deviation) or associated estimates of uncertainty (e.g. confidence intervals) |
| ☒ | ☐ | For null hypothesis testing, the test statistic (e.g. $F$, $t$, $r$) with confidence intervals, effect sizes, degrees of freedom and $P$ value noted<br>*Give P values as exact values whenever suitable.* |
| ☒ | ☐ | For Bayesian analysis, information on the choice of priors and Markov chain Monte Carlo settings |
| ☒ | ☐ | For hierarchical and complex designs, identification of the appropriate level for tests and full reporting of outcomes |
| ☒ | ☐ | Estimates of effect sizes (e.g. Cohen's $d$, Pearson's $r$), indicating how they were calculated |

*Our web collection on statistics for biologists contains articles on many of the points above.*

## Software and code

Policy information about availability of computer code

| | |
|---|---|
| Data collection | Microsoft Excel 2016, Image Lab Version 6.0.1 |
| Data analysis | GraphPad Prism 7.00 |

For manuscripts utilizing custom algorithms or software that are central to the research but not yet described in published literature, software must be made available to editors and reviewers. We strongly encourage code deposition in a community repository (e.g. GitHub). See the Nature Portfolio guidelines for submitting code & software for further information.

## Data

Policy information about availability of data

All manuscripts must include a data availability statement. This statement should provide the following information, where applicable:

- Accession codes, unique identifiers, or web links for publicly available datasets
- A description of any restrictions on data availability
- For clinical datasets or third party data, please ensure that the statement adheres to our policy

All data are included in the manuscript or in the Supplementary Materials and is available from the corresponding author on request.

# Field-specific reporting

Please select the one below that is the best fit for your research. If you are not sure, read the appropriate sections before making your selection.

☒ Life sciences ☐ Behavioural & social sciences ☐ Ecological, evolutionary & environmental sciences

For a reference copy of the document with all sections, see nature.com/documents/nr-reporting-summary-flat.pdf

# Life sciences study design

All studies must disclose on these points even when the disclosure is negative.

| | |
|---|---|
| Sample size | The sample size was based on preliminary experiments and on careful review of the literature. |
| Data exclusions | No data was excluded. |
| Replication | The results of the EBV IgG seropositivity assay showing that not every CALD patient is EBV positive was successfully reproduced by measuring a new, limited sample set of children with cerebral ALD. |
| Randomization | Patients were not randomized. Samples from participants were allocated into different groups based on predefined inclusion and exclusion criteria. CALD patients were defined by presence of Gadolinium-enhancing brain MRI lesions at the time of blood sampling. The control cohort consisted of participants with no diagnosis of disorders characterized by neuroinflammation and/or axonal degeneration. |
| Blinding | All samples were pseudonymized and the measurements were carried out blinded for the phenotype or treatment. |

# Reporting for specific materials, systems and methods

We require information from authors about some types of materials, experimental systems and methods used in many studies. Here, indicate whether each material, system or method listed is relevant to your study. If you are not sure if a list item applies to your research, read the appropriate section before selecting a response.

## Materials & experimental systems

| n/a | Involved in the study | |
|---|---|---|
| ☐ | ☒ | Antibodies |
| ☐ | ☒ | Eukaryotic cell lines |
| ☒ | ☐ | Palaeontology and archaeology |
| ☒ | ☐ | Animals and other organisms |
| ☐ | ☒ | Human research participants |
| ☒ | ☐ | Clinical data |
| ☒ | ☐ | Dual use research of concern |

## Methods

| n/a | Involved in the study | |
|---|---|---|
| ☒ | ☐ | ChIP-seq |
| ☒ | ☐ | Flow cytometry |
| ☒ | ☐ | MRI-based neuroimaging |

## Antibodies

| | |
|---|---|
| Antibodies used | mouse anti-CD20 (Thermo scientific, MS-340, 1:100); mouse anti-EBV-LMP (Dakocytomation, M0897, 1:60); mouse anti-ABCD1 (Euromedex ALD-1D6-AS, clone 2AL-1D6, 1:10.000); mouse anti-ß-Actin (Chemicon/Merck, MAB1501, 1:100.000) |
| Validation | According to the manufacturer´s website, the mouse anti-CD20 antibody is intended for qualitative immunohistochemistry on FFPE. In addition, we validated the antibody by using tonsil tissue as a positiv control. The mouse anti-EBV-LMP antibody, according to the manufacturer, can be used for labeling of FFPE. We validated this antibody by staining EBV positive B Cell lymphoma. The mouse anti-ABCD1 AB 1D6 was validated previously in human X-ALD fibroblasts lacking the ABCD1 protein and is routinely used in our lab (Wiesinger et, JBC 2013) . The mouse anti-ß-Actin AB MAB1501 is a highly published monoclonal antibody that has been validated for use in WB. |

## Eukaryotic cell lines

Policy information about cell lines

| | |
|---|---|
| Cell line source(s) | HEK-293: ATCC; EBV-immortalized B lymphocytes: Dr. Ann Moser, Kennedy Krieger Institute, Baltimore, USA and Prof. Florian Eichler, Harvard Medical School, Boston, USA |
| Authentication | HEK-293 cell line authentication was based on morphology; Authentication of EBV-immortalized B lymphocytes was based on morphology and FACS |
| Mycoplasma contamination | The cell lines were not tested for mycoplasma contamination. |

| Commonly misidentified lines (See ICLAC register) | *Name any commonly misidentified cell lines used in the study and provide a rationale for their use.* |
|---|---|

## Human research participants

Policy information about studies involving human research participants

| | |
|---|---|
| Population characteristics | B cell isolation:<br>Adult X-ALD patients: male, AMN status with clinical symptoms of axonopathy in the spinal cord but no signs of cerebral involvement (CALD) at brain MRI, median age = 38<br>Adult healthy controls: male, no diagnosis of disorders characterized by neuroinflammation and/or axonal degeneration, median age = 37<br>EBV seropositivity:<br>childhood CALD patients: children with MRI-confirmed CALD lesions before allogeneic stem cell transplantation, median age = 8; children controls: age-matched control group consisting of children with conditions unrelated to neuroinflammation including X-ALD, median age = 8 |
| Recruitment | For B cell isolation, recruitment of Austrian and German X-ALD patients occured through the patient organization ELA Germany. The EBV seropositivity testing was done in course of preparing patients for allogeneic stem cell transplantation and was retrospectively compared to values from healty control children. Thus, for this analysis, no active recruitment was done. |
| Ethics oversight | The study was approved by the Ethical Committee of the Medical University of Vienna (EK1462/2014 and EK1613/2019). |

Note that full information on the approval of the study protocol must also be provided in the manuscript.
